# Mean and variance heterogeneity loci impact kernel compositional traits in maize

**DOI:** 10.1002/tpg2.70131

**Published:** 2025-10-09

**Authors:** Yasser M. A. Ismail, Christopher Mujjabi, Marcus O. Olatoye, Stephen Gray, Alexander E. Lipka, Martin O. Bohn

**Affiliations:** ^1^ Department of Crop Sciences University of Illinois Urbana‐Champaign Illinois USA; ^2^ USDA‐ARS‐Forage Seed and Cereal Research Unit Prosser Washington USA; ^3^ Department of Agronomy Iowa State University Ames Iowa USA

## Abstract

Maize (*Zea mays*) kernel composition is critical for food, feed, and industrial applications. Improving traits such as starch, protein, oil, fiber, and ash requires understanding their genetic basis. We conducted genome‐wide association studies (GWAS) and variance genome‐wide association studies (vGWAS) analyses using 954 inbred lines from the USDA‐ARS North Central Regional Plant Introduction Station collection to identify loci influencing both trait means and variability. We detected 10 significant single nucleotide polymorphisms (SNPs) associated with five kernel traits, some of which colocalized with known genes such as *waxy1* and *gras7*. vGWAS uncovered additional loci not detected by standard GWAS, highlighting its value as a complementary tool. Genomic selection models, including ridge‐regression best linear unbiased prediction, reproducing kernel Hilbert space, and random forest, achieved moderate prediction accuracies (0.41–0.55), with parametric and semi‐parametric models showing less prediction bias. Although our dataset was derived from unreplicated genebank seed, key findings, particularly for protein and starch, were consistent with results from replicated field trials, supporting the utility of genebank‐derived high‐quality samples for initial genomic analysis. These results highlight the potential for using existing seed resources and high‐throughput phenotyping to identify candidate loci and prioritize traits for future replicated validation.

## Core Ideas

- Maize kernel composition is governed by a polygenic architecture, with few moderate‐effect quantitative trait locus (QTL) and many small‐effect loci.
- Both mean single nucleotide polymorphisms (mSNPs) and variance SNPs (vSNPs) contribute to phenotypic variation, often through pleiotropic effects.
- Several identified vSNPs and mSNPs colocalized with known candidate genes associated with kernel compositional traits.
- Variance genome‐wide association studies (vGWAS) serves as a powerful complementary tool to genome‐wide association studies (GWAS) for detecting novel loci and understanding phenotypic variability.
- Parametric, semi‐parametric, and nonparametric genomic selection (GS) models performed similarly in predicting kernel composition traits.
- This study highlights the value of integrating GWAS, vGWAS, and GS for genomic‐assisted breeding of maize nutritional quality traits.

## Plain Language Summary

Corn kernels provide protein, starch, and oil that are vital for food, animal feed, and fuel. These qualities differ among corn types and are shaped by genetics. We studied which parts of the genome affect not only average nutrient levels but also how consistent they are across plants. We also tested whether genetic information from DNA can predict these traits to guide breeding. Using a diverse set of corn lines and quick, nondestructive measurements, we found genome regions that influence both nutrient levels and consistency. Some regions affected several traits at once. DNA‐based predictions worked well, even with single seed samples. Analyzing existing seed collections with simple tools can give valuable insights, helping breeders save time and resources while improving corn for food and agriculture.

**Abbreviations**
BLUP: best linear unbiased predictor
GWAS: genome‐wide association studies
mSNPs: mean single nucleotide polymorphisms
NAM: nested association mapping
PVE: proportion of variation explained
QTL: quantitative trait locus/loci
RR‐BLUP: ridge‐regression best linear unbiased prediction
SNP: single nucleotide polymorphism
vGWAS: variance genome‐wide association studies
vQTL: variance QTL

## 1 INTRODUCTION

Maize (*Zea mays*) is a key staple grain in both developed and developing countries, composed of starch, protein, lipids, carotenoids, anthocyanins, and other essential nutrients (Baye et al., 2006). Targeted genetic improvements in maize kernel composition can help address hidden hunger, stunting, and undernutrition globally. Hidden hunger occurs when children lack essential vitamins and minerals, while undernutrition refers to insufficient nutrient absorption for growth (UNICEF, 2019). Stunting, caused by inadequate nutrition, repeated infections, and poor health, affects over 149 million children under five worldwide, leading to cognitive and physical impairments (UNICEF, 2024). Genetic improvements in maize composition can also provide industrial benefits, such as modifying starch content, lipids, and anthocyanin pathways to create specialty products (Cook et al., 2012). Traditionally, maize breeding focused on yield, but the rise of niche markets for specialty corns (e.g., Sweetcorn, waxy corn, Popcorn, silage corn, high‐oil corn, and Quality Protein Maize) and the demand for alternative feedstocks (e.g., starch variants and color pigments) have shifted breeding priorities toward kernel composition and quality (Scott et al., 2019).

In the past two decades, advances in quantitative and molecular genetics have broadened our understanding of the genetic basis and regulatory networks that underlie maize kernel composition. Maize germplasm with unique kernel composition characteristics was developed by using mutations affecting kernel composition and quality. The mutant *opaque2* (*o2*) increases the lysine content of the grain protein (Mertz et al., 1964); *linoleic acid1* (*ln1*) alters the fatty acid ratio of the grain oil (Poneleit & Alexander, 1965); and *waxy1* (*wx1*) (Lambert, 2001), *sugary1* (*su1*), *sugary enhancer* (*se*), and *shrunken2* (*sh2*) (Schultz & Juvik, 2004) change the starch composition of the grain.

Numerous quantitative trait locus (QTL) studies have characterized the genetic architecture of maize kernel compositional traits, revealing that these traits are controlled by multiple genes with small additive effects. Early work by Dudley et al. (1977) and later by Dudley and Lambert (1992), using the Illinois Long‐term Selection experiment, demonstrated that protein and oil content are governed by polygenic inheritance. This was further supported by Berke and Rocheford (1995), who used 200 F_2_S_1_ lines derived from a cross between Illinois High Oil (IHO) and Illinois Low Oil (ILO) strains to map QTL associated with protein, oil, and starch, identifying 16, 31, and 13 marker loci, respectively. Additional studies have employed diverse mapping populations—including Illinois High and Low Protein and IHO/ILO (Alrefai et al., 1995; Dudley et al., 2004; Goldman et al., 1993; Laurie et al., 2004; Wassom et al., 2008; Willmot et al., 2006), Beinongda high‐oil (Song & Chen, 2004; J. Zhang et al., 2008), Tropical high‐oil (Mangolin et al., 2004), Alexho single‐kernel high‐oil (Y. L. Li et al., 2009; Wang et al., 2010; Yang et al., 2012; Zheng et al., 2008), and Popcorn (Yanyang et al., 2008) lines to uncover QTL influencing kernel protein, oil, and starch content. More recently, H. Li et al. (2013) used genome‐wide association studies (GWAS) to identify 74 candidate loci associated with kernel oil concentration and fatty acid composition. Together, these findings provide a foundation for developing marker‐assisted and genomic selection (GS) strategies, as well as gene editing approaches, to enhance the nutritional and industrial quality of maize grain.

Variance genome‐wide association studies (vGWAS) are a more recent approach to identifying and mapping variance QTL (vQTL). vQTL contribute to trait variability that is usually undetected through standard biparental or association statistical mapping procedures (Forsberg & Carlborg, 2017; Rönnegård & Valdar, 2011; X. Shen et al., 2012). It has been argued that variance heterogeneity among genotypes can be partially explained by epistasis or gene‐by‐environment interactions (Brown et al., 2014; Forsberg & Carlborg, 2017; Young et al., 2018). Therefore, vQTL can provide insights into epistasis or phenotypic plasticity (Nelson et al., 2013; Young et al., 2018). Moreover, vGWAS can be a tractable approach to reduce the search space when assessing epistasis among markers (Brown et al., 2014; Wei et al., 2016).

Many studies have reported vQTL associated with diverse phenotypes in Drosophila (Ayroles et al., 2015; Mackay & Lyman, 2005), in mice (Corty et al., 2018; Nachman et al., 2003), and in humans (Topless et al., 2015; Wei et al., 2017; Yang et al., 2012; Young et al., 2018). In plants, vGWAS have been limited to a few species, including maize (Kusmec et al., 2017), Arabidopsis (Forsberg et al., 2015; X. Shen et al., 2012), and bread wheat (Hussain et al., 2020). The vGWAS approach facilitates detection of loci involved in the genetic control of environmental variation. It also allows loci mapping where incomplete LD between the causal polymorphism and the tested marker, multiple functional alleles, gene–gene, or gene‐by‐environment interactions lead to heterogeneous variance, rather than a mean difference between the genotype classes (Deng & Paré, 2011; Rönnegård & Valdar, 2011).

The North Central Regional Plant Introduction Station (NCRPIS) in Ames, IA, curates 20,283 *Zea* accessions, of which 74% can be requested for research (NCRPIS, 2021). In this experiment, we wanted to investigate whether it is possible to use these seeds to study the genetic basis and variation of kernel composition in maize. Even though it seems logical and straightforward to tap into this resource using high‐throughput phenotyping methods (e.g., near‐infrared spectroscopy), there are obvious limitations that must be considered carefully. Given that seed requests from the NCRPIS are limited to 100 kernels per accession obtained from a single nursery, our experiment was unreplicated. Replication allows the estimation of the experimental error, which enables the separation of the proper treatment effect from background noise, hence increasing the reliability and integrity of the experimental results (Girma & Machado, 2013). However, there are situations where experiments with treatment replication are logistically impossible due to high costs in time and financial resources and insufficient experimental units to replicate (Perrett & Higgins, 2006). Unreplicated experiments are not uncommon in agricultural research. For instance, early‐generation trials in breeding programs normally contain a large number of genotypes, and due to seed limitations unreplicated multilocation field trials are conducted. Therefore, a broad screening of genotypes using nonreplicated experimental designs is conducted to rank and select a subgroup of potentially high‐performing individuals for later advanced trials with replications (Gonçalves et al., 2013).

Due to the limited quantity of seed available per accession from the NCRPIS repository, we were restricted to a single near‐infrared (NIR) analysis per genotype, making independent replication infeasible without resorting to pseudo‐replication. Pseudo‐replication, as described by Hurlbert (1984), involves treating multiple measurements from a single experimental unit as independent observations, a method that can lead to misleading results due to the lack of genuinely independent responses to treatment, as noted by D. H. Johnson (2006). Additionally, pseudo‐replicated experiments often use artificially inflated degrees of freedom, resulting in a higher *F‐*statistic and increased risk of false positives (S. N. Johnson et al., 2016). To evaluate the quality and applicability of our unreplicated data, we compared the kernel compositional traits from our study with those from a replicated study by Renk et al. (2021), who examined a subset of 501 inbred maize lines from the Wisconsin Diversity (WiDiv) population across five different environments, each with two replications, to study the genetic control of traits like starch, protein, and oil using NIR spectroscopy. Their findings showed significant variation in kernel composition, primarily due to genetic and environmental factors. Notably, 275 of the 501 inbred lines examined by Renk et al. (2021) overlapped with our Ames Diversity panel subset. We utilized the Renk et al. (2021) replicated study results to determine the reliability of our data for genetic analysis, hypothesizing that strong positive correlations between phenotypic trait values in both studies would confirm the validity of our unreplicated study's results. The objectives of our study were to (i) determine the genetic architecture of the chemical composition of maize kernels, (ii) evaluate the potential of GS for nutritional crop improvement, and (iii) provide additional insights into natural variation in kernel composition by extending the standard GWAS to vGWAS.

**Core Ideas**

- Maize kernel composition is governed by a polygenic architecture, with few moderate‐effect quantitative trait locus (QTL) and many small‐effect loci.
- Both mean single nucleotide polymorphisms (mSNPs) and variance SNPs (vSNPs) contribute to phenotypic variation, often through pleiotropic effects.
- Several identified vSNPs and mSNPs colocalized with known candidate genes associated with kernel compositional traits.
- Variance genome‐wide association studies (vGWAS) serves as a powerful complementary tool to genome‐wide association studies (GWAS) for detecting novel loci and understanding phenotypic variability.
- Parametric, semi‐parametric, and nonparametric genomic selection (GS) models performed similarly in predicting kernel composition traits.
- This study highlights the value of integrating GWAS, vGWAS, and GS for genomic‐assisted breeding of maize nutritional quality traits.

## 2 MATERIALS AND METHODS

### 2.1 Germplasm and genomic data

A set of 954 diverse inbred lines obtained from the National Plant Germplasm System maize collection in Ames, IA, was used in this study (USDA Agricultural Research Service., 2024). The seed supplies for the accessions we received have been increased with standard genebank procedures (controlled pollination and individual plot harvesting) designed to minimize the chance of cross‐pollination or seed admixture. Available information about trueness‐to‐type or quality for accessions of this crop is provided through Germplasm Resources Information Network (GRIN) or by the crop curator. Genomic data for these lines, generated via genotyping‐by‐sequencing (Romay et al., 2013), are publicly available through the Maize Panzea directory on Cyverse. Raw single nucleotide polymorphism (SNP) data were initially filtered to remove entries with >80% missing data and minor allele frequency (MAF) < 0.01. Imputation was conducted chromosome‐by‐chromosome using BEAGLE 5.0 (Browning et al., 2018) with default settings. Post‐imputation, SNPs with MAF < 0.02 were excluded, resulting in a dataset of 264,428 SNPs across the 954 lines. Principal component analysis (PCA) was then performed using the full SNP dataset to assess genomic variation and population structure.

### 2.2 Phenotypic data collection

The kernel compositional analysis of maize inbred line samples was conducted using a DA 7200 Diode Array NIR Spectrometer from Perten Instruments. For each inbred line, 100 whole kernels were placed in a 75‐mm‐diameter open‐faced sample cup and scanned using the NIR spectrometer. For each sample, two readings were taken, and the sample was repacked between readings. Kernel compositional traits, including starch (STA), protein (PRO), oil (OIL), fiber (FIB), and ash (ASH), were estimated from the in‐built prediction equations and calibrations developed by Perten Instruments, using partial least squares regression (www.perten.com). The final phenotypic values for each sample were obtained by taking the mean of the two readings. This mean was used for all downstream analysis. We report the starch, protein, oil, fiber, and ash contents on a dry matter percentage weight basis. Density (DEN) values are reported at a 15% moisture level.

### 2.3 Statistical analysis

We used the interquartile range (IQR) method to identify and remove outliers for each trait. Observations more than 1.5 IQR below the first quartile (Q1) or above the third quartile (Q3) were considered outliers and excluded. This filtering removed 93 data points, leaving 861 observations for downstream phenotypic analysis. For each compositional trait, we calculated the mean, standard deviation (SD), minimum (Min), and maximum (Max). Pearson correlation coefficients were computed to assess relationships between kernel compositional traits. Due to the lack of replication per genotype, we did not estimate genotype effects directly. However, to evaluate the quality and informativeness of our unreplicated data, we conducted the following analyses. To assess differences in kernel compositional traits across broad maize subpopulations (Stiff Stalk, non‐Stiff Stalk, Tropical, Popcorn, and Sweetcorn), we fit a mixed linear model that included maize group (i.e., subpopulation) as a random effect:

(1) $$y_{ij}=\mu +G_{j}+\varepsilon_{ij},$$

where *y_ij_* is the phenotypic value of the *i*th genotype in the *j*th group, *μ* is the intercept, *G_j_* is the random effect of maize group [*G* ∼ *N*(0, **I** $\sigma_{G}^{2}$)], and *ε_ij_* is the residual error term. This model allowed us to estimate variance components attributable to group differences and generate best linear unbiased predictors (BLUPs) for each group. We did not include individual genotype effects in this model, as our objective was to characterize phenotypic variation at the subpopulation level, rather than to predict genotype‐specific effects. The model was fitted using the *lmer()* function from the lme4 package in R (Bates et al., 2015). BLUPs of group effects for each trait were estimated using the *coef()* function in R. Using the same model specification as Model 1, but with maize group treated as a fixed effect, we obtained the estimated means for each group. Pairwise comparisons between group means were conducted using Tukey's HSD test (*HSD.test()* function), with significance set at *α* = 0.05. Assumptions of homogeneity and normality of residuals were confirmed via *Q*–*Q* plots (Figure S1). Individual genotype‐level random effects and genomic relationship matrices were incorporated later in our GWAS and genomic prediction models (Sections 2.4, 2.7), where individual genetic variance and population structure were explicitly modeled.

We calculated Spearman rank correlations (Table 1) between our subpopulation BLUP means and those reported by Renk et al. (2021) to assess consistency in maize group performance rankings across studies. Additionally, we identified 275 genotypes common to both our study and Renk et al. (2021). We estimated BLUPs for these genotypes using the model described by Renk et al. across environments and replications, then performed Pearson correlation analysis to compare their BLUPs with our phenotypic means for each genotype. The correlation coefficients and associated *p*‐values are shown in scatter plots (Figure S2). All phenotypic data analyses were conducted using R version 4.2.1 (R Core Team, 2022).

**TABLE 1:** Best linear unbiased predictions (BLUPs) and observed means for kernel compositional traits across maize groups, and their correlations with corresponding literature‐reported values.

Group	*N*	Starch (%)		Protein (%)		Oil (%)		Fiber (%)		Ash (%)		Density (g/cm^3^)	
		BLUPs	Means	BLUPs	Means	BLUPs	Means	BLUPs	Means	BLUPs	Means	BLUPs	Means
Stiff Stalk	82	63.05	63.42a	12.11	11.92c	3.29	3.25b	1.33	1.34a	1.09	1.12a	1.30	1.30b
Non‐Stiff Stalk	88	63.08	63.10ab	12.13	12.06c	3.17	3.28b	1.27	1.28a	1.11	1.11a	1.31	1.30b
Popcorn	21	62.02	61.75bc	12.56	13.23a	2.79	2.43c	0.83	0.78bc	0.98	0.95b	1.34	1.34a
Sweet corn	39	60.89	60.26c	12.86	12.93a	4.41	5.25a	0.76	0.58c	0.99	0.99b	1.30	1.33a
Tropical	89	62.87	63.00ab	12.10	12.64ab	3.63	3.24b	1.04	0.95b	1.03	0.98b	1.32	1.33a
Unclassified	546	62.75	62.56b	12.42	12.48bc	3.38	3.52b	1.23	1.24a	1.09	1.11a	1.31	1.30b
Correlation^a^		0.94	0.94	0.82	0.82	0.14	0.14	−0.93	−0.93	−0.81	−0.77	NA	NA

*Note*: Means followed by the same letter within a trait are not statistically different according to Tukey's HSD (*p* < 0.05). NA, not available.

^a^ Spearman rank correlation coefficients between the observed and literature trait values for each maize group.

### 2.4 Genome‐wide association study

A GWAS was first performed to understand the genetic architecture of the traits of interest using the multi‐locus mixed model (MLMM) approach (Segura et al., 2012). GWAS analysis was performed using two types of the model implemented within the MLMM package. The MLMM method uses a stepwise mixed‐model regression procedure with forward selection and backward elimination. A total of 264,428 SNPs were used in the analysis across 954 accessions. The equation that describes the models is as follows:

(2) y=Sα+Xβ+Zu+e,

where **y** is the vector of phenotypes, **α** is the vector of the additive effects of the SNPs chosen by the model selection procedure, β is a vector of fixed effects accounting for the subpopulation structure, **u** is a vector of the random genetic background effects, and **e** is the vector of residual effects. The random effects **u** follow N(0, **K** $\sigma_{u}^{2}$), where **K** is the kinship matrix, and the residuals **e** follow N(0, **I** $\sigma_{e}^{2}$), with **I** as the identity matrix. **S** and **Z** are incident matrices of 1s and 0s relating y to **α** and **u**. **X** consists of a column of 1s with the remaining columns containing observed principal component (PC) values (Yu et al., 2006). The best MLMM model was selected using ExtBIC (Chen & Chen, 2008). The model was refitted using *lm*() to obtain *R* ^2^ estimates.

### 2.5 Variance heterogeneity mapping

Variance heterogeneity QTL were identified using the vGWAS R package (Hussain et al., 2020). The genome–phenotype association was carried out using the *vGWAS*() function. This function implements both double generalized linear model and hierarchical generalized linear model approaches to identify SNPs significantly associated with the variance of a trait (variance SNPs [vSNPs]). Genomic control (Devlin & Roeder, 1999) of the initial vGWAS results was performed using the *vGWAS.gc()* function. To correct for multiple testing, we applied a Bonferroni correction, setting the significance threshold at 1.89 × 10^−7^ (0.05/264,428 SNPs). While Bonferroni correction is conservative, particularly in high‐dimensional genomic analyses, we adopted this approach to reduce the likelihood of false positives given the known sensitivity of variance‐based tests. Our priority in this study was to identify robust and reproducible associations, especially given the exploratory nature of applying vGWAS to unreplicated seed bank material. We chose this threshold for its transparency and reproducibility, recognizing that future studies may complement this approach with less stringent alternatives such as false discovery rate control or permutation testing. The *vGWAS.variance()* function was used to estimate the proportion of variation explained (PVE) by both mean and variance for each vQTL and described in the following:

(3) $$V_{M}=pq\alpha^{2},$$

(4) $$V_{V}=pq\phi^{2},$$

where *V_M_* is the variance due to genetic effects on the mean, while *V_V_* is the variance due to heterogeneity between genotypes, *p* and *q* are the frequencies of the low and high variance alleles, and *α* and *ϕ* are the differences in the mean and standard deviation between the two homozygous genotypes, respectively (Hill & Mulder, 2010; X. Zhang & Hill, 2008).

### 2.6 A priori gene colocalization

A set of a priori candidate genes associated with maize grain quality, including *brittle endosperm2* (*bt2*), *gras7*, *linoleic acid1* (*ln1*), shrunken1 (*sh1*), *shrunken2* (*sh2*), and *waxy1* (*wx1*), was selected from the maize genetic database. To identify potential candidate genes underlying kernel compositional traits, QTL colocalization was assessed within a 100 kb window upstream and downstream of each gene using a custom R script. The script is available on GitHub at: https://github.com/BohnLabUIUC/AmesKernelComposition.

### 2.7 Genomic prediction

The goal of the genomic prediction analysis was to compare the performance of parametric approaches that only consider additive effects with nonparametric approaches based on their ability to capture cryptic non‐additive effects. Four GP models, that is, ridge‐regression best linear unbiased prediction (RR‐BLUP; parametric), reproducing kernel Hilbert space (RKHS; semi‐parametric), and random forest (RF; nonparametric), were used. The GP models were fitted simultaneously with each other using the fivefold cross‐validation approach. In fivefold cross‐validation, the dataset is randomly divided into five approximately equal subsets. In each fold, four subsets (the training set) are used to train the GS model, which then predicts genomic estimated breeding values for the remaining subset (the validation set). This process is repeated five times so that each subset serves once as the validation set. To ensure robustness, the entire fivefold cross‐validation procedure was repeated ten times with different random assignments of individuals to the five subsets in each cycle.

#### 2.7.1 Ridge regression BLUP

The mixed.solve() function in the RR‐BLUP package (Endelman & Jannink, 2012) was used to implement RR‐BLUP GS model (Meuwissen et al., 2001; Whittaker et al., 2000) and is described as follows:

(5) $$y=\mu +\sum_{m=1}^{p}Z_{m}u_{m}+e,$$

where ***y*** is the vector of phenotype values, μ is the vector of the intercept, **u** random effect term for the *m*th marker ∼*N*(0, $\sigma_{u_{m}}^{2}$), *p* is the number of genomic markers (where *p* > *n* [population size]), $Z_{m}$ is the *m*th column of the design matrix ***Z***, **u** is the vector of random marker effects associated with markers *m = *1… *p*. In the model, Var(**e**) = $\sigma^{2}$ (residual variance) and Cov(**u, e**) = 0. The RR‐BLUP subjects the predictions of all marker effects to the ridge parameter λ, which is estimated as $\frac{\sigma_{e}^{2}}{\sigma_{u_{m}}^{2}}$ (Endelman, 2011; Meuwissen et al., 2001) to alleviate the statistical challenge of having the number of markers exceed sample size (i.e., *p >* *> n*).

#### 2.7.2 Reproducing kernel Hilbert space

The RKHS models were implemented using the BLGR (Bayesian Linear Genomic Regression) package in R (Perez & De los Campos, 2014) with number of iterations and burns set to 12,000 and 2000, respectively. The model is described as follows:

(6) y=1μ+u+e,

where ***y*** is the vector of phenotype values; 1 is a vector of 1′s; μ is the intercept; **u** is the vector of random genotype effects ∼MVN (**0**, $K_{h}\sigma_{u}^{2}$), $K_{h}$ is the kernel entries matrix whose Gaussian kernel uses the squared Euclidean distance between genetic markers to estimate the level of relatedness between individuals, while *h* is the smoothing parameter that multiplies each element of $K_{h}$ by a constant; and **e** is the random residual vector ∼MVN (**0**, **I** $\sigma_{e}^{2}$). Priors of this Bayesian RKHS are p(μ,u,e) which are proportional to the product of density functions MVN (**0**, $K_{h}\sigma_{u}^{2}$) and MVN (**0**, **I** $\sigma_{e}^{2}$).

#### 2.7.3 Random forest

The random forest approach was implemented using the randomForest() function from the randomForest package in R (Breiman, 2001). Default settings were used for all parameters except for the number of trees (ntree), which was increased to 5000 to ensure model stability and improve prediction accuracy. The RF algorithm works by constructing an ensemble of decision trees using bootstrapped samples of the data and random subsets of predictors at each split, and then aggregating their predictions to improve accuracy and reduce overfitting. The model was trained using the marker data as predictors and the phenotypic values as the response variable.

### 2.8 Code and data availability

A repository containing all the scripts and documentation is available at: https://github.com/BohnLabUIUC/AmesKernelComposition.

## 3 RESULTS

### 3.1 Phenotypic analysis

**FIGURE 1:**
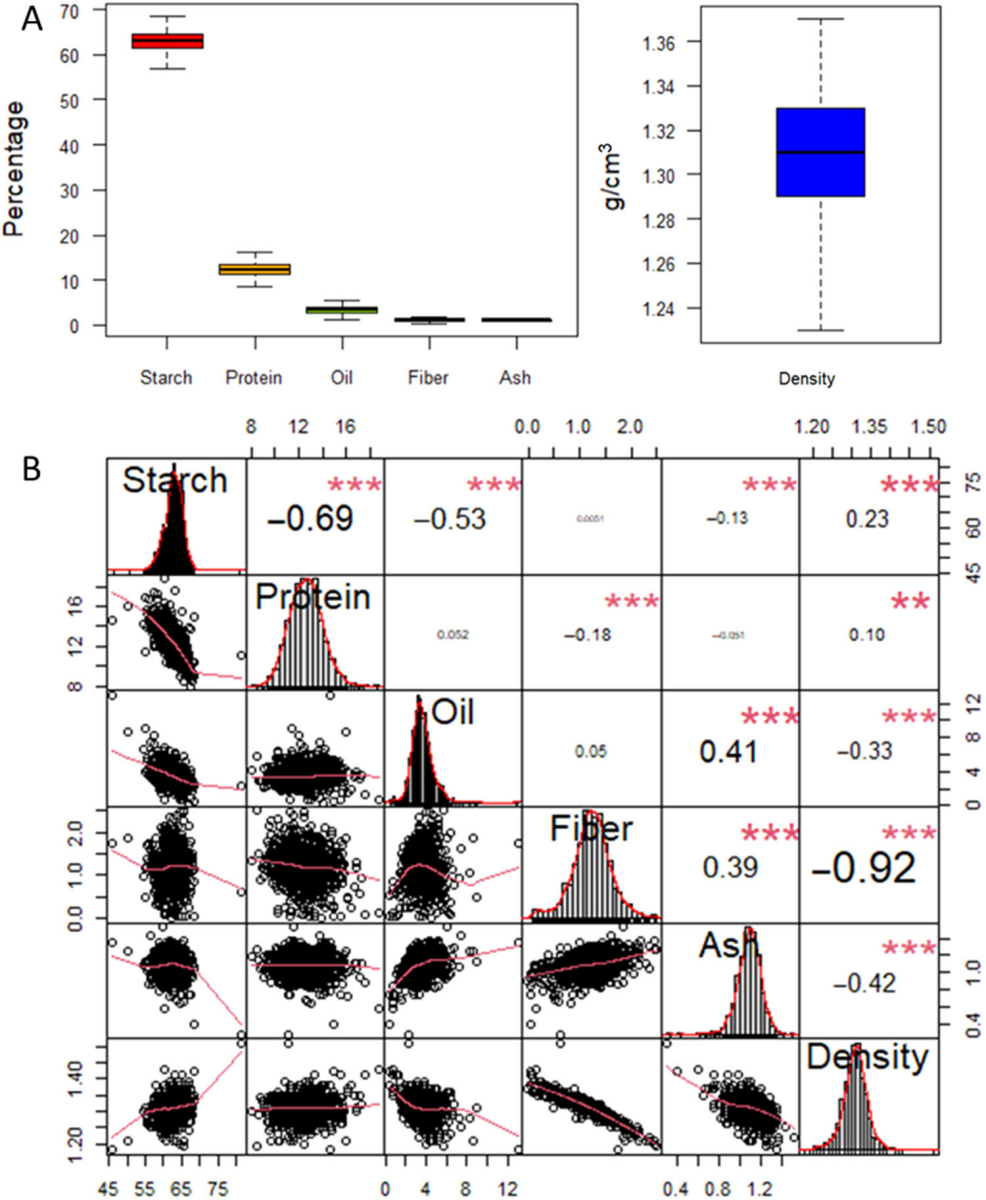
Distribution and pairwise relationships among grain composition and density traits. (A) Distribution of starch, protein, oil, fiber, ash, and density values across samples. (B) Pairwise trait relationships showing Pearson's correlation coefficients (above diagonal) and linear regressions (below diagonal). *, **, and *** indicate significance at the 0.05, 0.01, and 0.001 probability levels, respectively.

Phenotypic variation in kernel composition traits was evaluated in the diverse inbred panel using near‐infrared (NIR) spectroscopy. Descriptive statistics for starch (STA), protein (PRO), oil (OIL), fiber (FIB), ash (ASH), and kernel density (DEN) are summarized in Table S1. The distribution of each trait is shown in the boxplot in Figure 1A. Trait values ranged from 50.2% to 68.6% for STA, 8.0% to 17.5% for PRO, 0.4% to 9.0% for OIL, 0.03% to 2.5% for FIB, and 0.6% to 1.5% for ASH. Kernel density ranged from 1.2 to 1.4 g/cm^3^. The results of the correlation analysis among compositional traits are presented in Figure 1B. PRO was negatively correlated with STA (*r* = −0.69) and FIB (*r* = −0.18) and positively correlated with DEN (*r* = 0.11). STA was positively correlated with DEN (*r* = −0.23) and negatively correlated with OIL (*r* = −0.53) and ASH (*r* = −0.13). OIL showed significant negative correlations with both STA (*r* = −0.56) and DEN (*r* = −0.35), and a significant positive correlation with ASH (*r* = 0.45). FIB was positively correlated with ASH (*r* = 0.42) and strongly negatively correlated with DEN (*r* = −0.94).

**FIGURE 2:**
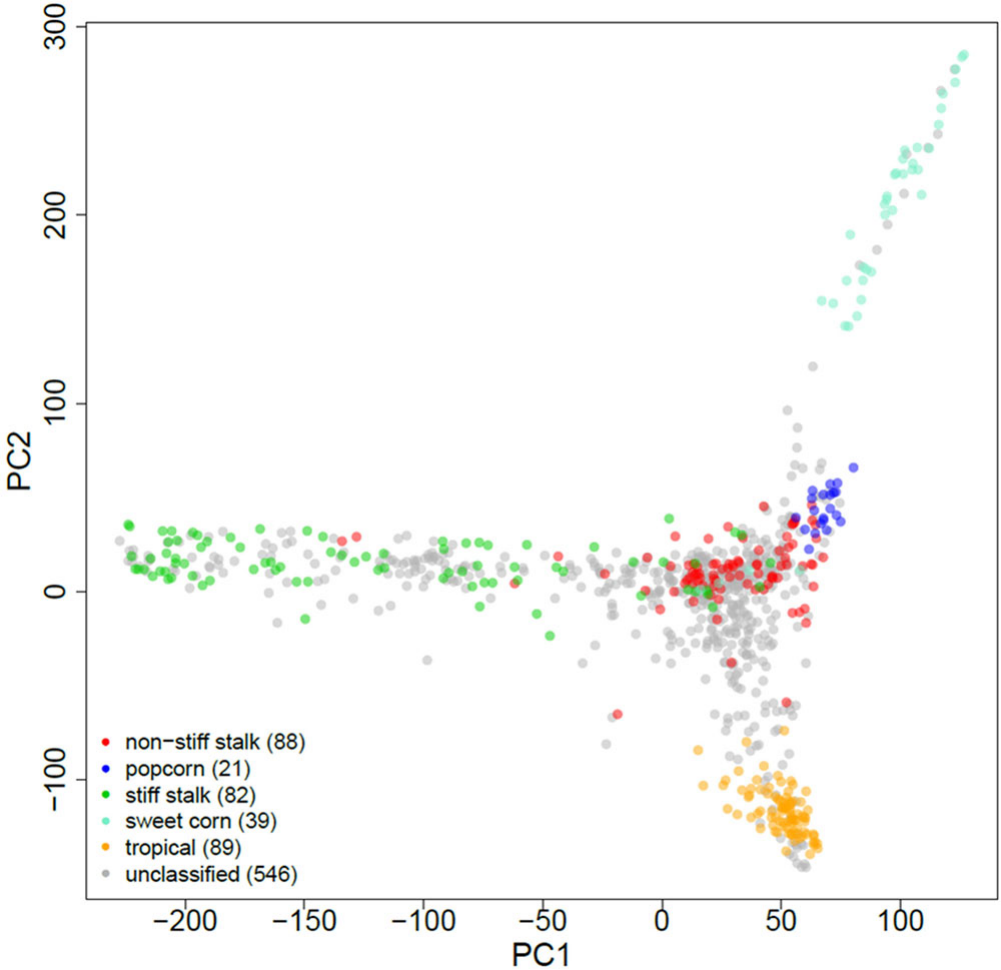
Principal component analysis of 954 maize inbred lines from the North Central Regional Plant Introduction Station (NCRPIS) germplasm bank. The *x‐* and *y‐* axes represent principal components 1 (PC1) and 2 (PC2), respectively. Colors indicate population structure.

PCA of the SNP data separated the inbred lines into Stiff Stalk, non‐Stiff Stalk, Popcorn, Sweetcorn, and Tropical maize groups, as shown in Figure 2. The PCA plot based on the first two PCs (PC1 and PC2) highlights clear genetic differentiation among these groups. In addition, phenotypic analysis revealed significant maize group effects for the kernel compositional traits, with group differences summarized in Table 1. Average starch content was highest in the Stiff Stalk group (63.4%) and lowest in the Sweetcorn group (60.3%). No significant differences in starch content were observed among the Stiff Stalk, non‐Stiff Stalk, and Tropical groups, nor between the Popcorn and Sweetcorn groups. Protein content was highest in the Popcorn group (13.2%) and lowest in the Stiff Stalk group (11.9%). Inbred lines from the Popcorn, Sweetcorn, and Tropical groups had significantly higher protein content than those from the Stiff Stalk and non‐Stiff Stalk groups, which did not differ significantly from each other. Oil content was significantly higher in the Sweetcorn group (5.3%) and lowest in the Popcorn group (2.4%). No significant differences in oil content were detected among the Stiff Stalk, non‐Stiff Stalk, and Tropical groups. Stiff Stalk and non‐Stiff Stalk inbreds had significantly higher fiber and ash content than the other groups, among which no significant differences were observed. Kernel density was significantly higher in the Popcorn, Sweetcorn, and Tropical groups compared to the Stiff Stalk and non‐Stiff Stalk groups.

Spearman rank correlation results between the trait BLUPs and means for the different maize groups in our study and those reported by Renk et al. (2021) are presented in Table 1. We observed strong and significant positive correlations for protein and starch BLUPs (*r* = 0.94 and 0.82, respectively) across the different maize groups. Oil BLUPs showed a moderate positive correlation (*r* = 0.14), while fiber and ash BLUPs were strongly negatively correlated (*r* = −0.93 and −0.77, respectively). Similar correlation patterns were observed when group means were analyzed instead of BLUPs. Additionally, Pearson correlation analysis was performed using compositional trait values for the 275 genotypes common to both studies. The resulting correlation coefficients are shown in the scatterplots in Figure S2. We observed strong positive correlations for protein (*r* = 0.60), oil (*r* = 0.39), and starch (*r* = 0.43) values. In contrast, fiber and ash values showed weak negative correlations (*r* = −0.11 and −0.04, respectively) between the two studies.

### 3.2 Genetic architecture of traits

#### 3.2.1 Main effect QTL

A diversity panel of 954 inbred maize lines genotyped with 264,428 SNPs was used to characterize the genetic architecture of kernel compositional traits through GWAS and variance heterogeneity mapping (vGWAS). Using the MLMM approach, GWAS identified a total of 10 significant SNPs associated with all six kernel traits: one SNP for ASH, one for DEN, two for FIB, two for PRO, one for STA, and three for OIL (Table 2 Figure S3). Among these, three SNPs were classified as large‐effect loci, each explaining more than 10% of the phenotypic variance (ASH: S8_145800871; FIB: S4_42805440; OIL: S6_104865219). The remaining SNPs each explained between 1% and 5% of phenotypic variation. Notably, SNP S9_23215076 (MAF = 0.02) was associated with both kernel density (DEN) and fiber (FIB), suggesting potential pleiotropic effects or closely linked loci.

**TABLE 2:** Maize kernel compositional traits associated with main effect SNPs obtained from a GWAS analysis.

Trait^a^	Marker	AES	PVE	MAF
STA	S3_25879786	0.90	2.00	0.04
PRO	S2_21008179	0.51	4.90	0.13
PRO	S4_222806611	0.27	2.20	0.23
OIL	S4_206444886	1.00	6.20	0.02
OIL	S5_196177600	0.67	5.10	0.04
OIL	S6_104865219	0.42	10.20	0.21
FIB	S4_42805440	0.31	10.20	0.04
FIB	S9_23215076	0.26	3.10	0.02
ASH	S8_145800871	0.13	12.50	0.03
DEN	S9_23215076	0.02	3.60	0.02

Abbreviations: AES, additive effect size; GWAS, genome‐wide association studies; MAF, minor allele frequency; PVE, proportion of variation explained; SNP, single nucleotide polymorphism.

^a^ Starch (STA), protein (PRO), oil (OIL), fiber (FIB), Ash (ASH), and density (DEN).

#### 3.2.2 Variance SNP

Variance genome‐wide association mapping was employed as a proxy approach to identify epistatic loci potentially contributing to the genetic architecture of maize kernel compositional traits. In total, 184 vSNPs were detected across all traits: 38 associated with ASH, 11 with DEN, 81 with FIB, 11 with OIL, seven with PRO, and 11 with STA. These vSNPs explained between 0.01% and 4.70% of the phenotypic variance in trait variability. The vSNP with the largest effect was S9_22098032 (DEN; 4.70%), followed by S7_174520093 (DEN; 3.50%), S9_45195731 (FIB; 3.10%), and S7_120567595 (FIB; 3.10%) (Table 3; Figure S4). Approximately half of the vSNPs explained more than 1% of the variance, while the remaining half explained less than 1%. Interestingly, the vGWAS approach identified a substantially greater number of associations than the GWAS approach, and no loci were shared between the vSNPs and the mean single nucleotide polymorphisms (mSNPs). In addition to identifying loci associated with trait variance, vGWAS also uncovered vSNPs that contributed to variation in trait means—quantified as the proportion of variance explained for the mean component (*V_m_* or PVE)—with some loci explaining up to 7.7% of mean phenotypic variation. Notably, several vSNPs exhibited nonzero effects on *V_m_* while having zero variance effects (*V_v_* = 0). In total, 50% (*n* = 92) of the vSNPs explained more than 1% of the variance in the mean.

**TABLE 3:** Maize kernel compositional traits associated variance SNPs from a vGWAS analysis.

Trait^a^	Marker	AES	*V_m_*	*V_v_*	MAF
PRO	S5_4669112	0.34	0.50	2.10	0.03
	S9_21820284	0.40	0.80	2.10	0.03
OIL	S8_153561670	0.20	0.40	4.10	0.05
FIB	S1_278034483	0.10	3.90	2.40	0.46
	S2_193208196	0.10	1.20	2.10	0.03
	S2_196411801	0.04	0.30	2.20	0.07
	S4_14950520	0.01	0.04	2.10	0.24
	S4_157839597	0.12	6.30	2.40	0.29
	S4_174003362	0.10	1.90	2.00	0.27
	S4_46934560	0.12	5.70	2.60	0.19
	S4_47513765	0.20	7.70	2.10	0.18
	S5_182134191	0.10	2.90	2.10	0.19
	S5_196179626	0.14	5.50	2.30	0.14
	S5_201069467	0.10	5.40	2.10	0.3
	S6_4438720	0.10	2.30	2.50	0.42
	S6_100083224	0.02	0.10	2.00	0.11
	S6_164557871	0.06	1.80	2.60	0.25
	S7_120558145	0.03	0.50	2.60	0.26
	S7_120567595	0.11	3.50	3.10	0.13
	S7_127739207	0.15	3.80	2.10	0.07
	S8_57119840	0.10	4.00	2.00	0.22
	S8_58885626	0.10	2.40	2.20	0.1
	S8_92276319	0.10	3.10	2.30	0.12
	S8_93794982	0.10	1.30	2.30	0.13
	S8_93800869	0.10	1.20	2.10	0.13
	S8_93802239	0.10	1.20	2.10	0.13
	S8_135051458	0.10	1.60	2.00	0.1
	S8_135059307	0.10	1.80	2.30	0.1
	S9_23215078	0.10	1.20	2.80	0.26
	S9_45195731	0.04	0.60	3.10	0.22
	S9_144639635	0.10	2.50	2.00	0.15
	S10_148536608	0.10	2.00	2.40	0.15
DEN	S7_174520093	0.01	1.50	3.50	0.04
	S9_22098032	0.00	0.01	4.70	0.06

Abbreviations: AES, additive effect size; MAF, minor allele frequency; SNP, single nucleotide polymorphism; *V_m_*, variance due to genetic effects on the mean; *V_v_*, variance due to heterogeneity between genotypes.

^a^ Protein (PRO), oil (OIL), fiber (FIB), and density (DEN).

### 3.3 Significant associations colocalized with candidate genes

This study identified several significant associations that colocalize with candidate genes previously implicated in the genetic control of kernel compositional traits in maize. A significant SNP marker (S9_23215076; MAF = 0.02; DEN:PVE = 3.6%; FIB:PVE = 3%) associated with both DEN and FIB colocalized with the maize *Waxy1* gene at about 53 kb away (Table 4). A main effect SNP (S6_104865219) associated with OIL colocalized with *ln1* at a distance of approximately 160 kb away. A significant vSNP (S9_23215078; MAF = 0.26; *V_m_* = 1.2%, *V_v_* = 2.8%; Table 4) associated with FIB also colocalized with the maize *Waxy1* gene at about 53 kb. A significant vSNP (S6_104976064; MAF = 0.02; *V_m_* = 0.16%; *V_v_* = 0.004%; Table 4) associated with ASH colocalized with *In1* at a distance of about 50 kb. In addition, a significant vSNP (S3_166434867; MAF = 0.20; *V_m_* = 0.01; *V_v_* = 0.05; Table 4) associated with STA colocalized with *gras7* at about 237 kb. Similarly, another vSNP (S3_166205478; MAF = 0.20; *V_m_* = 0.75; *V_v_* = 0.24; Table 4) associated with OIL colocalized with *gras7* at about 8 kb.

**TABLE 4:** Summary of SNPs associated with maize kernel compositional traits colocalized with a priori genes.

Trait^a^	Marker	Model	Chr	Position	MAF	Gene^b^	Maize_ID^c^	Proximity (kb)
OIL	S3_166205478	vGWAS	3	166,205,478	0.20	*gras7*	GRMZM2G013016	806
	S6_104865219	GWAS	6	104,865,219	0.21	*In1*	GRMZM2G169089	161
FIB	S3_217007766	vGWAS	3	217,007,766	0.10	*sh2*	GRMZM2G429899	502
	S9_23215076	GWAS	9	23,215,076	0.02	*wx1*	GRMZM2G024993	53
	S9_23215078	vGWAS	9	23,215,078	0.26	*wx1*	GRMZM2G024993	53
	S9_24197681	vGWAS	9	24,197,681	0.19	*wx1*	GRMZM2G024993	926
DEN	S9_23215076	GWAS	9	23,215,076	0.02	*wx1*	GRMZM2G024993	53

Abbreviations: Chr, chromosome; GWAS, genome‐wide association studies; MAF, minor allele frequency; SNP, single nucleotide polymorphism; vGWAS, variance genome wide association mapping.

^a^ Oil (OIL), fiber (FIB), and density (DEN).

^b^ *Waxy1* (*wx1*), *Inoleum 1* (*In1*), *shrunken 2* (*sh2*), and *gras 7* (gras7).

^c^ Maize gene identifier.

### 3.4 Genomic selection for kernel compositional traits

**FIGURE 3:**
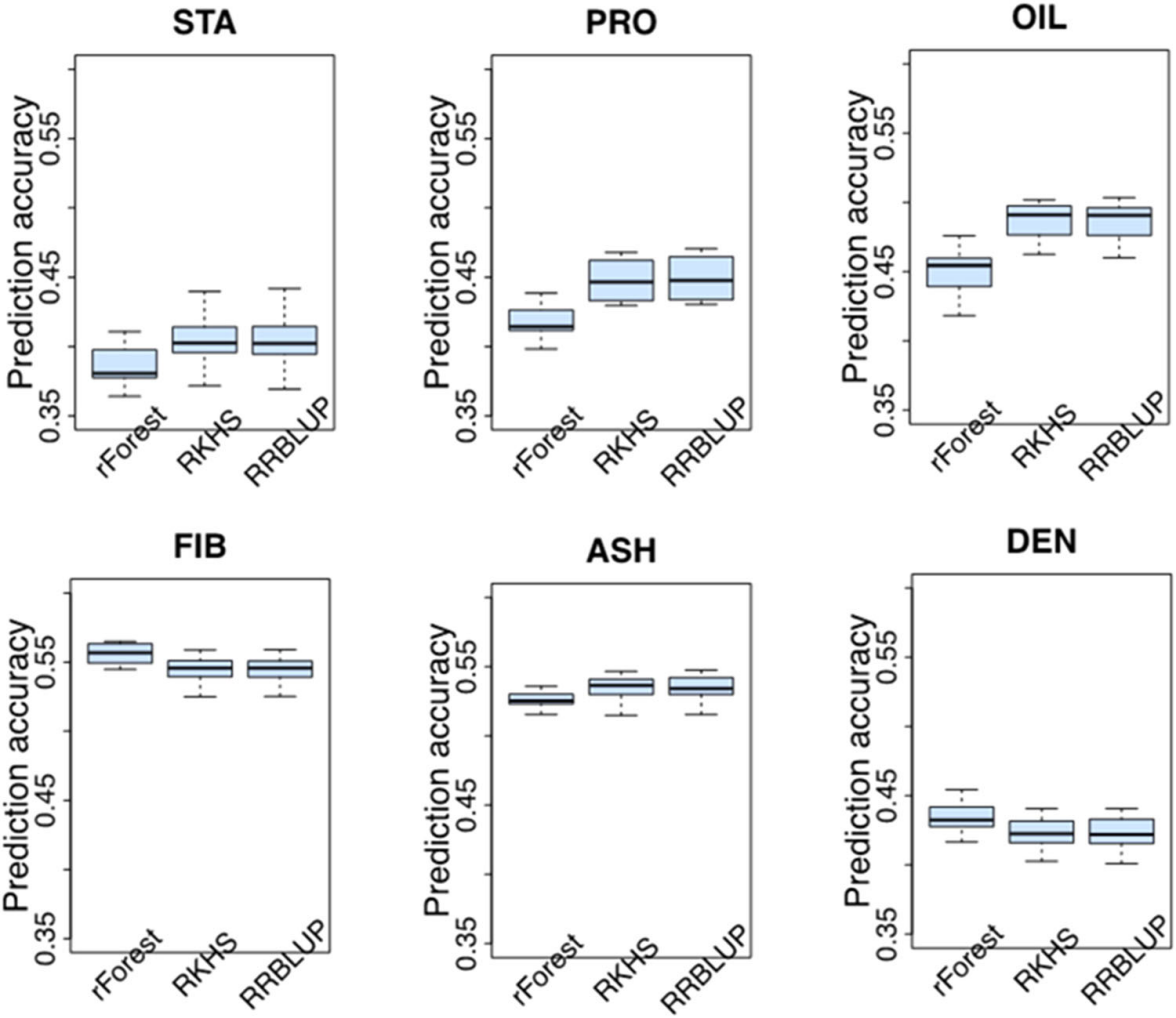
Comparison of prediction accuracy among genomic selection (GS) models. Boxplots in each panel showed the distribution of prediction accuracy values across 10 cycles of fivefold cross‐validation for random forest (rForest; nonparametric model), reproducing kernel Hilbert space (RKHS; semi‐parametric model), and ridge regression best linear unbiased prediction (RR‐BLUP; parametric model). ASH, ash; DEN, density; FIB, fiber; OIL, oil; PROT, protein; STA, starch.

**FIGURE 4:**
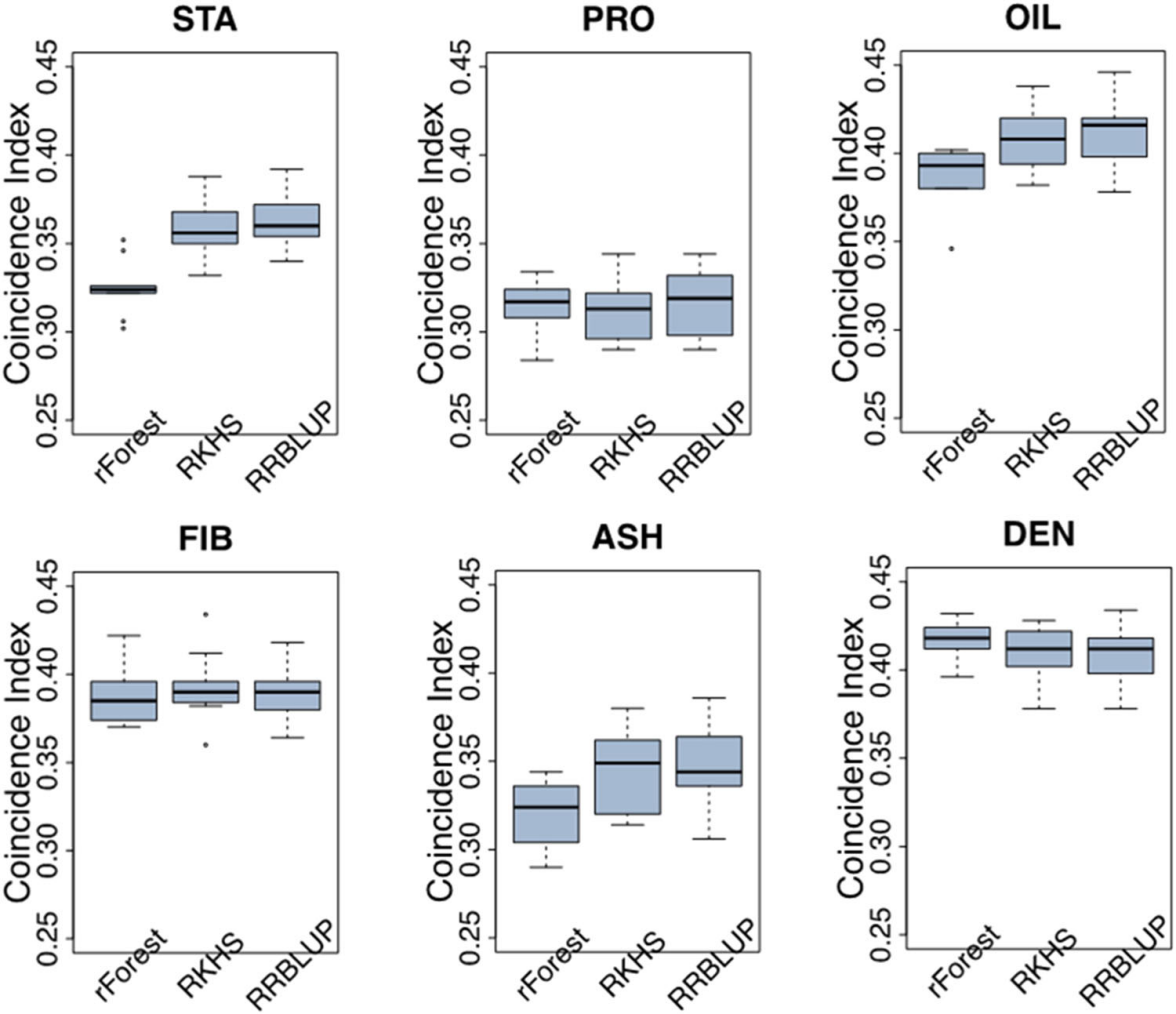
Comparison of coincidence index performance among genomic selection (GS) models. Boxplots in each panel showed the distribution of coincidence index values across 10 cycles of fivefold cross‐validation for random forest (rForest; nonparametric model), reproducing kernel Hilbert space (RKHS; semi‐parametric model), and ridge regression best linear unbiased prediction (RR‐BLUP; parametric model). ASH, ash; DEN, density; FIB, fiber; OIL, oil; PROT, protein; STA, starch.

Previous knowledge about the genetic architecture of a trait can help make informed decisions in breeding. In this study, we used representative parametric (RR‐BLUP), semi‐parametric (RKHS), and nonparametric (rFOREST) models to perform GS. Results indicated that the GS models performed similarly across all traits, with mean prediction accuracies for RR‐BLUP and RKHS being closely aligned (Figure 3; Table S2). Both models had the highest mean prediction accuracy for PRO, STA, and OIL. The largest difference in performance between rFOREST, RR‐BLUP, and RKHS was for OIL. Applying RR‐BLUP and RKHS increased prediction accuracy by 8%. The nonparametric GS model (rFOREST) had a slightly higher prediction accuracy for FIB (0.56) than RR‐BLUP (0.54) and RKHS (0.54). Similarly, GS models’ coincidence index performance (Figure 4; Table S3) was comparable to their prediction accuracy. In general, no single trait had the overall best performance across all traits. RR‐BLUP and RKHS yielded more reliable estimates of the genotype–phenotype relationship, as indicated by slope values closer to 1 and intercepts nearer to 0, compared to rFOREST (Tables S4 and S5).

## 4 DISCUSSION

Maize kernel composition is a quantitatively inherited trait controlled by both major‐effect and numerous minor‐effect genes. As a result, genetic improvement of kernel compositional traits through conventional breeding remains challenging, particularly given the negative correlations observed between grain yield and key compositional traits such as protein content. The development of molecular marker technologies has enhanced the efficiency and precision of selection in maize breeding programs. This study utilizes available seed from maize inbred accession in the USDA‐ARS NCRPIS collection for comprehensive kernel composition analysis. We analyzed 954 inbred lines to identify SNPs and candidate genes associated with kernel compositional traits, with the aim of expanding our understanding of their genetic architecture. Unlike previous studies that relied on replicated field experiments, our analyses were conducted using seed directly obtained from the seed bank. To validate the robustness of this approach, we compared our findings with those from replicated multi‐environment studies, assessing consistency in trait correlations and genetic associations.

### 4.1 Trait correlation analysis

The correlation analysis revealed both positive and negative relationships among kernel compositional traits (Figure 1B). In particular, starch content was negatively correlated with both oil and protein, consistent with previously reported phenotypic correlations in maize (Cook et al., 2012; Dudley et al., 2004; Vanous et al., 2019; Wassom et al., 2008). These strong correlations likely reflect underlying genetic relationships among traits and have long posed challenges for the simultaneous improvement of multiple kernel quality traits. Negative correlations—such as those observed between starch and protein—complicate breeding strategies aimed at increasing both traits concurrently. Such relationships are often attributed to pleiotropic gene action or linkage disequilibrium (Panthee et al., 2005; Simmonds, 1995). Supporting this, results from the Illinois Long‐term Selection Program demonstrated that starch, protein, and oil content are regulated by a complex genetic network characterized by strong pleiotropic interactions (Cook et al., 2012; Dudley et al., 2004; Wassom et al., 2008). These findings align with our correlation results and highlight the genetic constraints inherent in compositional trait improvement.

### 4.2 Variation in kernel composition across maize groups

To examine the variation in kernel composition across genetic backgrounds, we evaluated compositional trait differences among maize groups within the set of diverse maize used in this study. PCA of SNP data revealed clear population structure corresponding to known genetic differentiation among maize subpopulations. These patterns are consistent with previous analyses of the NCRPIS inbred lines (Pinho Morais et al., 2020; Romay et al., 2013; Tolley et al., 2021). Phenotypic analysis showed significant differences in kernel composition among the maize groups. As expected, inbred lines from the Sweetcorn group exhibited significantly lower starch content than all other groups. This is likely due to functional variants in endosperm genes involved in starch biosynthesis, such as *waxy1* (*wx1*), *sugary1* (*su1*), *sugary enhancer1* (*se1*), *brittle1* (*bt1*), *brittle2* (*bt2*), *shrunken2* (*sh2*), and *amylose‐extender1* (*ae1*), which promote the accumulation of sugars at the expense of starch (Tracy, 2001). These results align with those of Renk et al. (2021), who also reported significantly reduced starch in sweet corn lines. The Stiff Stalk and non‐Stiff Stalk groups exhibited the highest starch and lowest protein content. This likely reflects historical selection for increased endosperm starch content in these groups, which, due to the negative correlation between starch and protein, has led to a corresponding decrease in protein content (Dudley et al., 2004; Vanous et al., 2019; Wassom et al., 2008). Interestingly, the Sweetcorn group also showed significantly higher oil content compared to the other groups, in agreement with findings by Renk et al. (2021). Given that more than 85% of kernel oil is localized in the embryo (B. Shen & Roesler, 2017), and that germ size correlates with oil content and fatty acid composition (Ortíz‐Islas et al., 2019), these results suggest that sweet corn inbreds may possess relatively larger embryos than lines from other groups.

### 4.3 Phenotypic correlation analysis between studies

Given the use of unreplicated seed bank material in our study, we performed cross‐study comparisons to validate the reliability of our phenotypic data. Specifically, we compared kernel composition values obtained from our unreplicated analysis to those reported by Renk et al. (2021), who evaluated similar traits in a replicated, multi‐environment field experiment. Positive correlations between the two datasets would indicate that our measurements capture meaningful genetic signals despite the lack of replication. Spearman rank correlation analysis revealed consistent trends between the two studies for several kernel compositional traits across different maize groups. In particular, starch and protein content exhibited strong positive correlations, supporting the validity of our unreplicated phenotypic data. Pearson correlation analysis conducted on the 275 genotypes shared between the two studies further confirmed this pattern. Trait values for starch and protein showed strong and significant positive correlations, while oil content showed a moderate positive correlation. In contrast, ash and fiber content showed weak or negative correlations between the two datasets. These discrepancies in ash and fiber may be explained by differences in sampling methods, experimental design, and pollination strategy. Renk et al. (2021) used ground seed samples from open‐pollinated plots, while we analyzed whole kernels harvested from self‐pollinated plants grown in NCRPIS nurseries. Pollination method is known to influence kernel composition. For example, Letchworth and Lambert (1998) demonstrated that open‐pollinated kernels had higher starch and oil, but lower protein content compared to self‐pollinated kernels of the same genotype. Our observations mirror these findings: average starch and oil contents were higher in the open‐pollinated samples from Renk et al. (2021) (72.01% and 4.17%, respectively) than in our self‐pollinated samples (62.64% and 3.48%, respectively). Conversely, protein content was slightly higher in our samples (12.43%) than in those from Renk et al. (2021) (12.32%).

These differences may reflect not only xenia effects but also genotype‐by‐environment (G × E) interactions, which were present in the multi‐environment trial but absent in our seed bank study. Despite these potential sources of variance, the strong agreement observed for major compositional traits such as protein, starch, and oil supports the utility of unreplicated seed bank material for genetic analyses and validates the use of this approach for GWAS and vGWAS.

This study also paves the way for researchers to more directly leverage the NCRPIS germplasm resource to investigate kernel compositional traits using rapid, nondestructive, high‐throughput phenotyping tools such as near‐infrared spectroscopy (NIR), without the need to grow nurseries or conduct large‐scale field trials. In addition to generating reliable phenotypic data, this approach enables the collection of spectral data that can support fast and cost‐effective selection decisions through breeding tools such as phenomic prediction and selection. Weiß et al. (2022) compared the predictive abilities of GS and phenomic selection (PS) using NIR spectra for 400 genotypes and found comparable prediction accuracies for a range of agronomic traits. These results underscore the potential of our phenotyping strategy to support PS‐based screening directly within diversity panels, enabling more efficient use of genetic resources for breeding without the logistical burden of field trials.

### 4.4 GWAS identifies moderate‐effect loci associated with kernel composition

Our GWAS analysis identified several loci of small to moderate effect associated with kernel compositional traits in maize. These results are consistent with findings from the maize nested association mapping (NAM) population, where kernel composition traits were also shown to be polygenically controlled by numerous loci of modest effect (Cook et al., 2012). Although the number of significant associations detected in our study was lower than in NAM‐based studies, the effect sizes identified are likely more representative of the underlying genetic architecture due to our relatively large sample size (*N* = 954). This minimizes the risk of inflated effect estimates commonly observed in smaller populations, a phenomenon known as the Beavis effect (Beavis, 1998).

The number of loci detected in our study falls within the range reported in previous QTL mapping efforts focused on maize kernel composition (Clark et al., 2006; Dudley, 2008; Dudley et al., 2004; Goldman et al., 1993, 1994; Laurie et al., 2004; Séne et al., 2001; Wassom et al., 2008). However, our GWAS results revealed fewer associations than studies using the maize NAM population, likely due to the increased mapping power and controlled population structure of NAM. The NAM panel, consisting of approximately 5000 recombinant inbred lines across 25 families, offers enhanced resolution and detection power, as well as improved precision in estimating marker effects. Similar differences in mapping power between diversity panels and NAM designs have been demonstrated in sorghum (Bouchet et al., 2017).

Another factor potentially limiting our ability to detect associations is the confounding effect of population structure. In GWAS, population structure can bias association signals when trait variation is correlated with genetic relatedness (Myles et al., 2009). Although we applied mixed linear models to control for population structure and kinship, overcorrection can occur when traits are strongly associated with group membership, leading to a loss of true signals and an increased rate of false negatives. This phenomenon has been demonstrated in sorghum by Olatoye et al. (2020), who showed that even advanced models like MLMM can miss genuine associations when population structure is overly adjusted for.

Although the number of detected associations in our study was modest, the results are consistent with prior studies of kernel composition traits in maize and reflect the complex, polygenic nature of these traits. Moreover, our results highlight the value of large, diverse association panels and underscore the importance of carefully accounting for population structure to maximize discovery while minimizing bias.

### 4.5 vGWAS identifies novel loci associated with variance in kernel composition

To complement our GWAS analysis, we performed variance genome‐wide association studies (vGWAS) to identify loci influencing phenotypic variability rather than trait means. While GWAS targets main‐effect QTL that shift the average phenotype, vGWAS detects vQTL that regulate trait variability, often reflecting gene‐by‐gene (epistatic) or gene‐by‐environment interactions (Brown et al., 2014; Forsberg & Carlborg, 2017; Hussain et al., 2020; H. Li et al., 2020; Murphy et al., 2022). Our vGWAS analysis uncovered a substantial number of significant associations, many of which were not detected by standard GWAS. Notably, there was no overlap between the SNPs identified by vGWAS (vSNPs) and those identified by GWAS (mSNPs), suggesting that vGWAS captures a distinct and complementary set of loci. This contrasts with findings from H. Li et al. (2020), who reported that approximately 6.2% of vSNPs overlapped with mSNPs in their study of grain composition traits.

Many of the vSNPs detected in our study contributed to both mean (*V_M_*) and variance (*V_V_*) components of phenotypic variation. *V_M_* values ranged from 0% to 8%, indicating that these loci had measurable effects on both average trait values and variability across genotypes. Interestingly, several vSNPs showed nonzero effects on *V_M_* while having no detectable contribution to *V_V_* (i.e., *V_V_* = 0), highlighting the utility of vGWAS in revealing cryptic loci that might be missed by GWAS due to trait‐by‐environment heterogeneity or model assumptions.

To our knowledge, this represents one of the most extensive applications of combined GWAS and vGWAS to dissect maize kernel composition. The integration of both approaches led to the identification of more loci than any single maize genome‐phenome mapping study for compositional traits to date. These findings reinforce the value of vGWAS as a complementary strategy for discovering novel loci involved in complex trait architecture and suggest that vQTL mapping can enhance our understanding of trait stability, variability, and underlying genetic interactions.

Epistatic interactions among vQTL have been reported for grain composition in cereals (H. Li et al., 2020; Hussain et al., 2020). Variance heterogeneity mapping has also been proposed as a strategy to reduce the computational search space for epistasis detection (Hussain et al., 2020). While effective, this approach may miss interactions among loci that do not exhibit variance heterogeneity. Future work could explore alternative dimensionality reduction strategies, such as sure independence screening, to improve the power and scalability of higher order epistasis detection (Mathew et al., 2018).

### 4.6 Colocalization with known genes underlying kernel composition

To assess the biological relevance of the associations identified in this study, we examined the genomic positions of significant SNPs for colocalization with previously characterized genes involved in kernel composition. Several loci identified through both GWAS and vGWAS were found in close proximity to known functional genes, supporting the validity of our findings. A notable SNP (S9_23215076) significantly associated with both kernel density and fiber content colocalized with the *wx1* at approximately 53 kb. This SNP explained 3.6% of the variance in density and 3.0% in fiber (Table 4). Given the strong negative correlation observed between density and fiber, the dual association of this marker suggests a possible pleiotropic role or linkage to a regulatory region affecting both traits. *Waxy1* encodes granule‐bound starch synthase I, which is critical for amylose biosynthesis in the endosperm, and has been previously implicated in starch‐related trait variation in maize. In addition, a significant vSNP (S3_166434867) associated with starch content colocalized with *gras7*, a gene involved in gibberellin signaling, at a distance of approximately 237 kb. Another vSNP (S3_166205478), significantly associated with oil content, also colocalized with *gras7*, but at a distance of only 8 kb. The repeated association of *gras7* with multiple kernel traits may point to a broader regulatory role, potentially through hormonal control of resource allocation between the endosperm and embryo.

These colocalization results further support the robustness of our association analyses and suggest that a subset of the identified SNPs are located within or near functionally relevant genes. Moreover, the presence of shared loci across compositional traits is consistent with previous reports of pleiotropic effects in the genetic control of grain quality (Cook et al., 2012; Panthee et al., 2005; Simmonds, 1995). The identification of these candidate genes provides a foundation for future functional validation studies and marker‐assisted selection efforts targeting compositional improvement in maize.

### 4.7 Genomic selection performance and implications for breeding

To evaluate the potential of GS for improving kernel composition traits, we compared the predictive performance of three classes of models: parametric (RR‐BLUP), semi‐parametric (RKHS), and nonparametric (rFOREST). Consistent with previous findings (Heslot et al., 2012; Olatoye et al., 2019), no single model consistently outperformed the others across all traits, highlighting the importance of model selection based on specific trait architectures. While overall prediction accuracies were moderate to high, we also assessed model calibration using regression slope and intercept values from the relationship between observed and predicted phenotypes. RR‐BLUP and RKHS models produced slope values closer to 1 and intercepts nearer to 0, suggesting more accurate and less biased predictions compared to RF, which showed greater deviation from these ideal values (Tables S4 and S5). These results indicate that despite its flexibility, the rFOREST model may underperform in accurately estimating trait ranges, particularly when extrapolating beyond the training data. Such bias has practical implications for breeding, where selection decisions are made based not only on accuracy but also on the reliability and scale of predictions. Similar limitations have been reported for other machine learning algorithms, such as support vector regression (Olatoye et al., 2019), reinforcing the importance of using multiple performance metrics when evaluating GS models.

Together, the findings from our GWAS, vGWAS, and genomic prediction analyses provide a more comprehensive understanding of the genetic architecture underlying kernel composition traits in maize. The identification of novel loci through vGWAS, alongside the evaluation of GS model performance using multiple metrics, underscores the importance of integrating diverse analytical approaches to dissect complex traits. These insights not only enhance our ability to identify genetic contributors to trait means and variability but also inform practical breeding strategies aimed at improving compositional quality while maintaining predictive reliability. Building on this foundation, future efforts should focus on validating key loci in replicated field trials and exploring the incorporation of vQTL information into genomic prediction models to further optimize selection for both trait performance and stability.

## AUTHOR CONTRIBUTIONS

**Yasser M. A. Ismail**: Resources; writing—original draft; writing—review and editing. **Christopher Mujjabi**: Data curation; formal analysis; writing—original draft; writing—review and editing. **Marcus O. Olatoye**: Formal analysis; writing—original draft; writing—review and editing. **Stephen Gray**: Investigation; writing—review and editing. **Alexander E. Lipka**: Methodology; supervision; writing—review and editing. **Martin O. Bohn**: Conceptualization; funding acquisition; writing—review and editing.

## CONFLICT OF INTEREST STATEMENT

The authors declare that they have no competing interest.

## Supporting information



Supplementary Material

Supplementary Material
