# Supplementary material for: Mean and variance heterogeneity loci impact kernel compositional traits in maize
Source: Plant Genome. 2025 Oct 9;18(4):e70131. doi: 10.1002/tpg2.70131 (PMC12511846; doi:10.1002/tpg2.70131)
Supplement: Supplementary file 1 [file TPG2-18-e70131-s001.docx]

**SUPPLEMENTARY FIGURES**

Figure S1. Quantile-quantile (Q-Q) plots of the six chemical compositional traits for the mixed effects model. The plots show the distribution of the observed values against the expected normal distribution.

Figure S2. Scatter Plots showing the Pearson correlation analysis of the kernel compositional trait values for the 275 common genotypes between our study and Renk et al (2021) replicated study.

Figure S3. Genome wide association mapping for maize maize kernel traits using MLMM model. Genome physical positions of loci associated with (A) protein (PRO), (B) fibre (FIB), (C) starch (STA), (D) ash (ASH), (E) density (DEN), and (F) oil (OIL). *A priori* candidate genes that colocalize with significant SNPs are noted in italicized texts on broken lines.

Figure S4. Variance genome wide association mapping for maize maize kernel traits. Genome physical positions of variance SNPs associated with (A) protein (PRO), (B) fibre (FIB), (C) starch (STA), (D) ash (ASH), (E) density (DEN), and (F) oil (OIL). A priori candidate genes that colocalize with significant SNPs are noted in italicized texts on broken lines.
