# Supplementary material for: Mean and variance heterogeneity loci impact kernel compositional traits in maize
Source: Plant Genome. 2025 Oct 9;18(4):e70131. doi: 10.1002/tpg2.70131 (PMC12511846; doi:10.1002/tpg2.70131)
Supplement: Supplementary file 2 [file TPG2-18-e70131-s002.docx]

**SUPPLEMENTARY TABLES**

**Table S1.** Descriptive statistics of the kernel compositional traits

| Trait^a^ | Mean ± SD | Minimum | Maximum |
| --- | --- | --- | --- |
| STA | 62.64 ± 2.58 | 50.20 | 68.56 |
| PRO | 12.43 ± 1.46 | 7.99 | 17.51 |
| OIL | 3.48 ± 1.06 | 0.38 | 8.97 |
| FIB | 1.19 ± 0.39 | 0.03 | 2.49 |
| ASH | 1.09 ± 0.12 | 0.29 | 1.53 |
| DEN | 1.31 ± 0.03 | 1.18 | 1.51 |

^a^	Ash (ASH), density (DEN), fiber (FIB), oil (OIL), protein (PRO), and starch (STA).

**Table S2.** Summary of prediction accuracy estimates by genomic selection models.

| Trait*^a^* | rForest*^b^* | RRBLUP*^c^* | RKHS*^d^* |
| --- | --- | --- | --- |
| STA | 0.39±0.02 | 0.40±0.02 | 0.40±0.02 |
| PRO | 0.42±0.01 | 0.45±0.02 | 0.45±0.01 |
| OIL | 0.45±0.02 | 0.49±0.01 | 0.49±0.01 |
| FIB | 0.56±0.01 | 0.54±0.01 | 0.54±0.01 |
| ASH | 0.53±0.01 | 0.53±0.01 | 0.54±0.01 |
| DEN | 0.43±0.01 | 0.42±0.01 | 0.42±0.01 |

^a^	Ash (ASH), density (DEN), fiber (FIB), oil (OIL), protein (PRO), and starch (STA).

^b^	Random Forest

^c^	Ridge regression best linear unbiased predictions

^d^	Reproducing kernel Hilbert space

**Table S3.** Summary of coincidence index estimates by genomic selection models.

| Trait*^a^* | rForest*^b^* | RRBLUP*^c^* | RKHS*^d^* |
| --- | --- | --- | --- |
| STA | 0.32±0.02 | 0.36±0.02 | 0.36±0.02 |
| PRO | 0.32±0.01 | 0.32±0.02 | 0.31±0.02 |
| OIL | 0.39±0.02 | 0.41±0.02 | 0.41±0.02 |
| FIB | 0.39±0.02 | 0.39±0.02 | 0.39±0.02 |
| ASH | 0.32±0.02 | 0.35±0.03 | 0.35±0.02 |
| DEN | 0.42±0.01 | 0.41±0.02 | 0.41±0.02 |

^a^	Ash (ASH), density (DEN), fiber (FIB), oil (OIL), protein (PRO), and starch (STA).

^b^	Random Forest

^c^	Ridge regression best linear unbiased predictions

^d^	Reproducing kernel Hilbert space

**Table S4.** Summary of slope estimates by genomic selection models.

| Trait^a^ | rForest^b^ | RRBLUP^c^ | RKHS^d^ |
| --- | --- | --- | --- |
| STA | 1.16±0.05 | 0.93±0.05 | 0.96±0.05 |
| PRO | 1.33±0.06 | 1.01±0.05 | 1.04±0.05 |
| OIL | 1.19±0.05 | 1.01±0.04 | 1.03±0.04 |
| FIB | 1.23±0.02 | 0.99±0.03 | 1.01±0.03 |
| ASH | 1.34±0.05 | 1.01±0.03 | 1.03±0.03 |
| DEN | 1.20±0.04 | 0.99±0.04 | 1.02±0.04 |

^a^	Ash (ASH), density (DEN), fiber (FIB), oil (OIL), protein (PRO), and starch (STA).

^b^	Random Forest

^c^	Ridge regression best linear unbiased predictions

^d^	Reproducing kernel Hilbert space

| Trait^a^ | rForest^b^ | RRBLUP^c^ | RKHS^d^ |
| --- | --- | --- | --- |
| STA | -10.02±2.88 | 4.41±2.9 | 2.55±2.96 |
| PRO | -4.08±0.69 | -0.19±0.58 | -0.51±0.57 |
| OIL | -0.66±0.16 | -0.02±0.14 | -0.10±0.14 |
| FIB | -0.28±0.03 | 0.01±0.03 | -0.01±0.03 |
| ASH | -0.36±0.05 | -0.01±0.03 | -0.03±0.03 |
| DEN | -0.27±0.05 | 0.01±0.05 | -0.03±0.05 |

**Table S5.** Summary of intercept estimates by genomic selection models.

^a^	Ash (ASH), density (DEN), fiber (FIB), oil (OIL), protein (PRO), and starch (STA).

^b^	Random Forest

^c^	Ridge regression best linear unbiased predictions

^d^	Reproducing kernel Hilbert space
